# Inflammation and Coronary Microvascular Dysfunction in Autoimmune Rheumatic Diseases

**DOI:** 10.3390/ijms20225563

**Published:** 2019-11-07

**Authors:** Elisabetta Zanatta, Claudia Colombo, Gianpiero D’Amico, Thomas d’Humières, Carlo Dal Lin, Francesco Tona

**Affiliations:** 1 Department of Medicine, Padova University Hospital, 35128 Padua, Italy; elisabettazanatta86@gmail.com; 2 Department of Cardiac, Thoracic, Vascular Sciences and Public Health, Padova University Hospital, 35128 Padua, Italy; claudia.colombo@studenti.unipd.it (C.C.); gianpiero.damico@hotmail.it (G.D.); griska3@yahoo.it (C.D.L.); 3 Department of Cardiovascular Physiology, AP-HP, Henri-Mondor Teaching Hospital, 94010 Créteil, France; thomasdhumieres@hotmail.fr

**Keywords:** autoimmune diseases, inflammation, coronary microcirculation

## Abstract

Autoimmune rheumatic diseases (ARDs) form a heterogeneous group of disorders that include systemic lupus erythematosus (SLE), systemic sclerosis (SSc), rheumatoid arthritis (RA), idiopathic inflammatory myopathies (IIMs), and systemic vasculitis. Coronary microvascular dysfunction (CMD) is quite common in patients with ARDs and is linked to increased cardiovascular morbidity and mortality. Inflammation plays a crucial role in the pathogenesis of both accelerated atherosclerosis and CMD in ARDs, especially in patients affected by SLE and RA. In this regard, some studies have highlighted the efficacy of immunosuppressants and/or biologics in restoring CMD in these patients. By contrast, the role of inflammation in the pathogenesis of CMD-SSc appears to be much less relevant compared to endothelial dysfunction and microvascular ischemia, with calcium-channel blockers providing some benefits. Few studies have endeavored to assess the occurrence of CMD in IIMs and systemic vasculitis, thus warranting further investigations. The present review summarizes the current evidence on the occurrence of CMD in ARDs, focusing on the role of inflammation and possible therapeutic approaches.

## 1. Introduction

Autoimmune rheumatic diseases (ARDs) form a heterogeneous group of disorders characterized by multiple organ involvement stemming from systemic inflammation and a dysregulation of the immune system with subsequent tissue damage. ARDs include systemic lupus erythematosus (SLE), systemic sclerosis (SSc), rheumatoid arthritis (RA), inflammatory idiopathic myopathies (IIMs), and systemic vasculitis. Accelerated atherosclerosis and coronary microvascular dysfunction (CMD) are the main causes of cardiovascular (CV) involvement in these patients, leading to increased cardiovascular morbidity and mortality [1].

**Figure 1:**
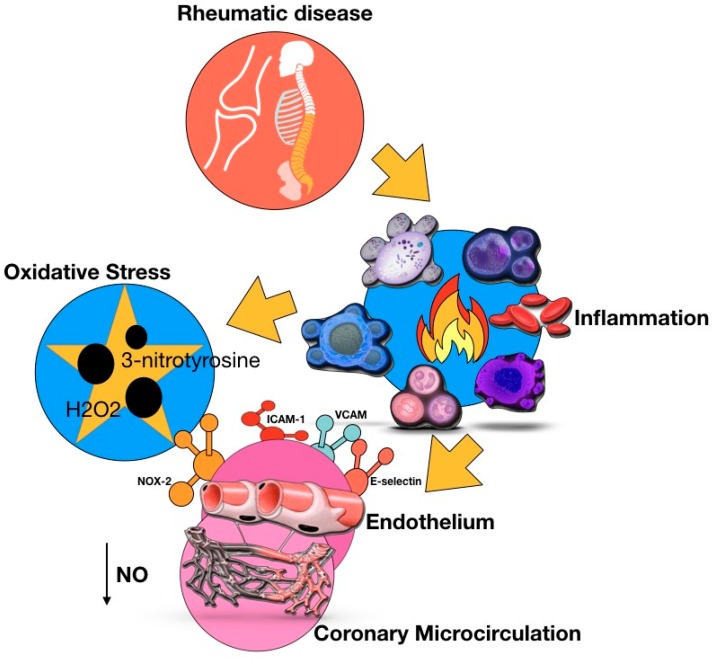
Systemic inflammation in autoimmune rheumatic diseases induces inflammatory endothelial activation and oxidative stress, which affect the coronary microvascular function. Systemic inflammation leads to the enhanced endothelial expression of adhesion molecules, such as ICAM-1, VCAM, and E-selectin. Inflammatory activation upregulates NOX2 in endothelial cells, which results in oxidative stress, increased levels of H_2_O_2_, eNOS uncoupling, decreased nitric oxide bioavailability, and formation of 3-nitrotyrosine.

In the literature pertaining to the CV implications in ARDS, much attention was initially given to the role of inflammation in inducing accelerated atherosclerosis—especially in SLE and RA—whereas the occurrence of CMD has only been more recently investigated. Endothelial dysfunction is thought to be an early event in the pathogenesis of both macro- and microvascular cardiac involvement in ARDs [2]. Decreased bioavailability of nitric oxide (NO) and increased production of reactive oxygen species (ROS) appear to be the nexus between pro-inflammatory molecules (e.g., tumor necrosis factor-α, interleukins (IL) 1β and 6) to endothelial dysfunction in these patients (Figure 1) [3]. A monocyte dysregulation with IL-1β production and the expansion of CD4+ CD28 null T cells, a subset of CD4+ lymphocytes with strong cytotoxic activity, are also proven molecular mechanisms involved in CMD-ARDs [4].

The coronary microcirculation comprises pre-arterioles (diameter 100–500 μm) and arterioles (diameter < 100 μm) and is of the utmost importance in determining myocardial perfusion as it accounts for 80% of the global blood flow resistance in the heart [5]. CMD reflects the inability of the cardiac microcirculation to appropriately dilate to meet myocardial oxygen demand because of functional impairment or structural damage of the coronary microcirculation. In patients affected by ARDs with normal epicardial coronary arteries, CMD is quite common and responsible for an increased risk of CV events and death, independently from traditional CV risk factors [6]. Considering healthy subjects, Vaccarino et al. demonstrated that in asymptomatic dizygotic twins, the presence of CMD (assessed by positron emission tomography (PET)) is associated with a systemic inflammatory response, defined by increased concentrations of C-reactive protein (CRP), intracellular adhesion molecule-1, and IL-6 [7]. In the context of ARDs, coronary microvascular disease appears due to several mechanisms that have more or fewer implications depending on the underlying disease. As mentioned earlier, there is a direct effect of chronic inflammation that leads to vasculopathy via endothelial activation. Moreover, there is a non-direct effect of chronic autoimmune activation that may lead to myocarditis, with diffuse interstitial edema, ultimately leading to microvascular ischemia [8].

Knowing the strong link between CMD and cardiovascular mortality in ARDs, it is crucial to diagnose cardiac involvement as early as possible and identify effective therapeutic strategies.

## 2. Imaging for Microvascular Dysfunction Screening

CMD can be detected by both non-invasive and invasive techniques. The coronary flow reserve (CFR) is the ratio between maximal (i.e., hyperemic) and baseline coronary blood flow, and a CFR ≤ 2.5 indicates CMD [9]. The assessment of CFR by standard transthoracic echocardiography (TTE) with adenosine or dipyridamole infusion is a validated method to investigate microvascular dysfunction in the absence of epicardial coronary stenosis that is widely performed in ARDs [10,11]. Perfusion defects can be assessed more accurately by contrast-enhanced cardiac magnetic resonance imaging (MRI) and stress perfusion MRI, which allow the quantification of the myocardial perfusion reserve index [12].

Single-photon emission computed tomography (SPECT) is another validated imaging modality to detect microvascular impairment. SPECT is a nuclear imaging technique performed at baseline or after stress, which reveals the differences in the distribution of the radionuclide (i.e., thallium-201) and, therefore, the relative blood flow to the different regions of the myocardium [13]. However, the only imaging modality able to measure absolute myocardial blood flow—thus allowing a direct and precise CMD assessment—is positron emission tomography (PET) with 18 F-fluorodeoxyglucose [13]. Furthermore, myocardial PET is a valuable tool for detecting and monitoring myocardial inflammation [13,14,15,16]. In fact, PET/MRI seems particularly promising for nuclear cardiology, in particular, with regard to inflammatory cardiac diseases, such as sarcoidosis [17]. Therefore, PET/MR represents a promising diagnostic approach for all cardiac diseases characterized by inflammation and microvascular impairment in the near future.

Microvascular impairment can also be quantified invasively by coronary angiography with the determination of the index of myocardial resistance using an intracoronary pressure wire at baseline conditions and during hyperemia [18].

## 3. Systemic Lupus Erythematosus

Systemic lupus erythematosus is a heterogeneous ARD that occurs predominantly in females and causes chronic inflammation in multiple organs. Cardiac manifestations (e.g., coronary artery disease (CAD) and myocarditis) are the leading cause of morbidity and mortality. SLE is associated with an accelerated atherosclerotic process, and it has been reported that women affected by SLE carry a 7.5-fold increased risk of developing CAD versus healthy controls [19,20]. Although there have been reports of a 50-fold increased risk of myocardial infarction among SLE women aged 35–44 years [21], the actual prevalence of subclinical heart disease in SLE remains unknown as it greatly depends on the diagnostic method used. Furthermore, traditional CV risk factors cannot fully explain the increased CV risk observed in patients with SLE, who often suffer from angina in the presence of a normal coronary angiogram. In this regard, Hirata et al. found that CFR was significantly lower in young (i.e., premenopausal) SLE women vs. controls, suggesting an impairment of the vasomotor tone and demonstrating an altered coronary microvascular function [22]. This evidence was corroborated by Yılmaz et al., who also found that CFR correlated directly with total antioxidant status and inversely with serum CRP levels, suggesting a prominent role for oxidative stress and inflammation in the pathogenesis of CMD in SLE [23]. In another small cohort of lupus patients with chest pain and non-obstructive CAD on coronary computed tomography angiography (CCTA), adenosine stress CMR was able to identify microvascular perfusion abnormalities in 44% of patients vs. 0% of healthy controls [24]. The authors concluded that chest pain in patients affected by SLE is mainly linked to microvascular coronary dysfunction and, thus, myocardial ischemia. This finding was further confirmed in studies conducted using SPECT and PET as imaging techniques [16,25]. Sandhu et al. recently investigated 20 female SLE patients with chest pain who underwent stress CMR and CCTA at baseline and after 5 years [26]. At follow up, the majority of patients had persistent angina, and nearly half had similar or worsened myocardial perfusion consistent with microvascular dysfunction without obstructive CAD.

While the link between symptomatic patients (i.e., chest pain) and CMD is well established, the data among non-symptomatic patients are still scarce. Recent studies on SLE patients without known heart involvement or CV symptoms reported an interesting association between troponin elevation, strain rate diminution (TTE), native T1 and T2 augmentation (MRI), aortic stiffness augmentation (pulse wave velocity), and myocardial inflammation/interstitial and perivascular fibrosis on histology [8,27]. These findings highlight the early subclinical heart involvement, resulting from endothelial inflammation and auto-immune activation.

All these findings show that CMD is quite common in SLE and represents a major cause of persistent chest pain in the absence of obstructive CAD in these patients. CMD and accelerated atherosclerosis alike appear to stem the inflammatory burden. Thus, the efficacy of corticosteroids and immunosuppressants in CMD-SLE warrants further investigation [23].

## 4. Systemic Sclerosis

Systemic sclerosis is a complex autoimmune connective tissue disease (CTD) characterized by immune dysregulation, microvascular damage, and fibrosis [28,29]. Functional endothelial impairment of the small vessels occurs early in the disease and manifests in the peripheral circulation as Raynaud’s phenomenon (RP). There is plenty of evidence supporting the microvascular origin of primary myocardial involvement in SSc [13], one of the main determinants of prognosis in these patients. As in other organs, the microangiopathic damage precedes the deposition of extracellular matrix and fibrosis, which may explain why the left ventricular (LV) diastolic dysfunction cannot be detected in the early “vascular phase” of myocardial involvement. In fact, one study performed SPECT in patients with SSc and other CTDs, which revealed reversible perfusion abnormalities in about half of SSc patients, suggesting cold-induced ischemia, i.e., “RP of the heart” [30]. Subsequently, Mizumo et al. demonstrated that the cardiac RP detected by myocardial contrast TTE is a strong predictor of future LV dysfunction [31].

Several studies with CFR measurement by TTE with adenosine infusion have found that CMD is detectable in about 50–60% of clinically asymptomatic scleroderma patients [32,33,34]. A cardiac MRI study found that 97% of patients had sub-endocardial perfusion defects and a lack of association between microvascular damage or myocardial fibrosis with atherosclerosis [15]. Moreover, we recently uncovered a strong association between CMD detected by coronary flow reserve and microvascular damage (i.e., avascular score) detected at nailfold video capillaroscopy, confirming the systemic nature of the microvascular impairment in SSc [34].

As in other microvascular manifestations of SSc (e.g., pulmonary arterial hypertension and digital ulcers), the main pathogenetic mechanisms underlying primary myocardial involvement seem to be an imbalance between mediators of vasoconstriction and vasodilation, resulting in ischemic damage. In these settings, calcium channel blockers (CCBs), such as nifedipine, have been shown to improve myocardial perfusion assessed by SPECT and prevent the future onset of LV diastolic dysfunction in scleroderma [13,35]. Moreover, a recent study conducted on the European database highlighted that both CCBs and low-dose acetylsalicylic are associated with a lower incidence of distinct primary myocardial disease manifestations in SSc [36].

On the other hand, a possible additional role of inflammation in CMD-SSc has been hypothesized recently. Conventional cardiac MRI has detected the presence of focal and diffuse myocardial fibrosis and, to a lesser extent, myocardial inflammation even in SSc with no cardiac symptoms [37]. Mavrogeni et al. demonstrated that silent myocarditis may be diagnosed in SSc by cardiac MRI using the Lake Louise criteria: T2 ratio, early (EGE), and late gadolinium-enhanced (LGE) images [38,39].

Moreover, SSc patients showed significantly expanded the extracellular volume and larger areas of T1 abnormality than those identified at conventional CMR, which likely reflects a combination of low-grade inflammation and diffuse myocardial fibrosis [37]. As a consequence, few studies in recent years have reported that immunosuppressants may delay the progression of cardiac damage, albeit with evidence gathered only from patients with concomitant myocarditis [40]. This concomitant toxicity of inflammation seems much more present as it pertains to severe patients with cardiovascular symptoms and elevated troponin or NT-pro-BNP. Endomyocardial biopsies performed in 25 symptomatic SSc patients found fibrosis and inflammation in 24. Moreover, the extent of fibrosis and inflammation correlated strongly with the prognosis [41].

These data point to a continuum between disease onset with microcirculation remodeling and low-grade ischemia, to diffuse myocardial fibrosis and inflammation induced by microvascular rarefaction and immune activation. Early subclinical microvascular heart impairment seems detectable, but aggressive vasodilator and immunosuppressive regimens warrant further investigation in these asymptomatic patients.

## 5. Rheumatoid Arthritis

Rheumatoid arthritis is linked to an increased risk of CV events. Large epidemiological studies have suggested that while traditional CV risk factors may be crucial, they alone cannot account for the added risk in RA patients [42]. The inflammation-driven atherogenesis may contribute to the increased CAD risk in RA patients [43], as corroborated by the proven efficacy of the interleukin-1 receptor antagonist Anakinra in improving endothelial, coronary and aortic functions, as well as the LV myocardial deformation and twisting in patients with RA and CAD [44]. However, it bears noting that RA patients carry a twofold increased risk of developing and dying from heart failure versus individuals without RA, even after adjusting for CAD [45].

Although the excess CV risk in RA is not fully understood, several mechanisms are believed to be responsible for endothelial dysfunction in these patients: genetic factors, immune dysregulation, inflammation, metabolic disturbances, use of steroid and anti-inflammatory drugs.

Toutouzas et al. reported that RA and diabetic patients presented similar rates of ischemic alterations at dobutamine stress TTE, significantly higher vs. controls [46]. Nevertheless, patients with RA less often than controls presented an epicardial obstructive disease, supporting the role of microvascular impairment in the genesis of ischemia and myocardial damage in RA [46].

Several studies have investigated CMD in patients with RA. Recently, Amigues et al. found that 30% of RA patients showing no clinical CV disease had a pathological (i.e., <2.5) myocardial flow reserve (MFR) assessed by PET-CT [47]. Moreover, higher IL-6 levels were significantly associated with lower MFR, whereas the use of tumor necrosis factor inhibitors was associated with higher MFR. As described earlier in SLE, the chronic inflammatory state of RA seems to contribute to both the accelerated atherosclerosis and CMD. In this regard, a group of RA patients without traditional CV factors showed a lower CFR combined with an increased thickness of the intima-media in [42]. Interestingly, these patients presented a well-controlled disease activity of RA, suggesting a subclinical chronic inflammation as well.

Altogether, these studies suggest that CMD and accelerated atherosclerosis share similar pathogenetic mechanisms in RA. Moreover, currently available data suggest that achieving remission in RA does not necessarily ensure the reversal of microvascular endothelial dysfunction. Since the treat-to-target is a cornerstone of the management of patients with RA, these findings suggest that restoring CMD may be considered as an adjunctive potential therapeutic target in RA. The demonstrated effect of some biologic therapies on CMD-RA could represent a key point in choosing biological drugs in RA patients, considering their high CV risk [44,47].

## 6. Systemic Vasculitis and Idiopathic Inflammatory Myositis

Systemic vasculitis refers to a heterogeneous group of autoimmune disorders characterized by vessel inflammation. Anti-neutrophil cytoplasmic antibody (ANCA)-associated vasculitis (AAVs) mainly include granulomatosis with polyangiitis (GPA), eosinophilic granulomatosis with polyangiitis (EGPA), and microscopic polyangiitis. AAVs affect small vessels, and, therefore, the cardiac impairment is intrinsically linked to a dysfunction of the coronary microcirculation. Perhaps because the microvascular origin is implicit in the classification of AAVs [48], there are no specific studies in the literature focused on investigating a coronary microvascular impairment in these patients. Cardiac involvement appears to be more frequent and severe in EGPA—and especially ANCA-negative—patients in whom it represents the main cause of first-year and overall death [49]. Hazebroek et al. found ECG and echocardiography abnormalities in 62% of EGPA and 46% of GPA patients linked to increased CV and all-cause deaths after a mean follow-up of 53 ± 18 months [50]. In the same study, additional CMR raised the prevalence of cardiac involvement to 66% in EGPA and 61% in GPA patients. Data from a cohort of 297 patients (including ANCA-associated vasculitis, non-ANCA-associated vasculitis, connective tissue disorders, arthritis, sarcoidosis) showed the highest prevalence of cardiac involvement in patients with ANCA-associated vasculitis, with LGE at CMR more frequently found in the EGPA subgroup (67%). The LGE pattern was usually present in the subendocardial segments of the entire LV circumference, sometimes in the mid-myocardial layer, and very rarely in the epicardial layer [51]. Though the significance of LGE in patients with ANCA-associated vasculitis and no cardiac symptoms remains unclear, a report by the French Vasculitis study group showed that immunosuppressants improved or normalized LGE in about 50% of patients with EGPA/GPA and other evidence of cardiomyopathy. Moreover, the aforementioned improvement correlated significantly to the absence of new cardiac complications during the follow-up [52]. These findings suggest that inflammation and autoimmunity may play an important role in the pathogenesis of cardiac involvement in ANCA-associated vasculitis, whose origin is microvascular.

As it pertains to large vessels vasculitis (i.e., Takayasu’s vasculitis and giant cells arteritis), the involvement of small vessels is very rare, and hence, CMD is very uncommon. Behar et al. reported a case of an Asian female affected by Takayasu’s vasculitis and refractory ischemic chest pain caused by CMD [53].

Microvascular dysfunction studied by CFR highlighted abnormalities in coronary microcirculation in Kawasaki disease. A reduced CFR was reported in medium and giant coronary aneurysms, and in stenotic lesions even of mild to intermediate severity. Therefore, abnormalities in the coronary microcirculation and epicardial lesions may occur concomitantly in these patients [54].

Finally, myocardial perfusion defects caused by CMD have been reported in patients with Behçet’s disease [55,56].

Idiopathic inflammatory myopathies (IIMs) are a group of chronic, autoimmune conditions characterized by the infiltration of inflammatory cells into skeletal muscles [57]. The most common are dermatomyositis, polymyositis, necrotizing autoimmune myopathy, and sporadic inclusion body myositis. Myocarditis is the more frequent cardiac involvement detectable in symptomatic patients with IIMs and occurs in up to 30% of patients at autoptic studies [58]. However, advanced echocardiographic techniques (i.e., tissue doppler imaging and speckle tracking) found that subclinical systolic dysfunction of both ventricles is detectable in about 50% of patients with IIMs, including those deemed in remission [59,60,61]. Although this may suggest a microvascular origin to the cardiac involvement in these patients, no specific studies have investigated CMD in IIMs so far.

## 7. Conclusions

Coronary microvascular disease is common in patients with ARDs, leading to increased CV risk. Inflammation is relevant in the pathogenesis of CMD-ARDs, especially in patients affected by SLE and RA, and, therefore, a more aggressive therapeutic approach with immunosuppressants and biologics might prove useful in these patients to prevent diastolic and systolic dysfunction. By contrast, autoimmunity and inflammation seem much less involved in CMD-SSc, whose treatment to date consists of vasodilators and low-dose aspirin. The occurrence of CMD has been less investigated in systemic vasculitis and IIMs, thus warranting further studies. The early identification of CMD and a more precise clarification of the pathogenetic mechanisms involved in CMD-ARDs is paramount in these patients to define the best therapeutic strategy and avoid major CV events.
